# Association of genetic polymorphisms of PCSK9 with type 2 diabetes in Uygur Chinese population

**DOI:** 10.1186/s12872-022-02710-w

**Published:** 2022-06-22

**Authors:** Meng-Meng Wang, Chen-Fei Lu, Shi-qi Yan, Bao-Zhu Wang, Gulinazi Yesitayi, Yong-Liang Tian, Xiang-Ma, Yi-Tong Ma

**Affiliations:** Department of Cardiology, First Affiliated Hospital of Xinjiang Medical University, Urumqi, 830011 People’s Republic of China

**Keywords:** PCSK9, Type 2 diabetes, Gene polymorphisms

## Abstract

**Background:**

PCSK9 gene expression is associated with biological processes such as lipid metabolism, glucose metabolism, and inflammation. In the present study, our primary objective was to assess the association between the single-nucleotide polymorphisms in the PCSK9 gene and type 2 diabetes in Uygur subjects, in Xinjiang, China.

**Methods:**

We designed a case–control study including 662 patients diagnosed with T2DM and 1220 control subjects. Four single-nucleotide polymorphisms (rs11583680, rs2483205, rs2495477 and rs562556) of PCSK9 gene were genotyped using the improved multiplex ligation detection reaction technique.

**Results:**

For rs2483205, the distribution of genotypes, dominant model (CC vs CT + TT), overdominant model (CC + TT vs CT) showed significant differences between T2DM patients and the controls (*P* = 0.011 and *P* = 0.041 respectively). For rs2495477, the distribution of genotypes, the dominant model (AA vs GA + GG) showed significant differences between T2DM patients and the controls (*P* = 0.024). Logistic regression analysis suggested after adjustment of other confounders, the differences remained significant between the two groups [for rs2483205 CC vs CT + TT: odds ratio (OR) = 1.321, 95% confidence interval (CI) 1.078–1.617, *P* = 0.007; CC + TT vs CT: OR = 1.255, 95% CI 1.021–1.542, *P* = 0.03; for rs2495477 AA vs GA + GG: OR = 1.297, 95% CI 1.060–1.588, *P* = 0.012].

**Conclusion:**

The present study indicated that CT + TT genotype and CT genotype of rs2483205, as well as GA + GG genotype of rs2495477 in PCSK9 gene were associated with an increased risk of type 2 diabetes in the Uygur population in Xinjiang.

**Supplementary Information:**

The online version contains supplementary material available at 10.1186/s12872-022-02710-w.

## Introduction

Type 2 diabetes mellitus (T2DM) is a complex metabolic disease mainly characterized by hyperglycemia arising from insulin resistance and/or insufficient insulin secretion [1]. Long-term chronic hyperglycemia can lead to multiple system damage and failure, such as heart, eyes, kidneys, nerves, and blood vessels [2]. Survey data show that the prevalence of T2DM has been rapidly rising worldwide and is projected to grow to 440 million by 2030 [3]. T2DM has become one of the serious public health problems. T2DM is the result of the interaction between genetic and environmental factors, a large number of studies have revealed that multiple single nucleotide polymorphisms (SNPs) are related to the development of T2DM [4, 5].

Proprotein convertase subtilisin/kexin type 9 (PCSK9) is a secreted protein that is mainly expressed in the liver, small intestinal epithelial cells, neural tissue, and kidney cells [6]. The function of PCSK9 is thought to increase the amount of low-density lipoprotein cholesterol (LDL-C) in the blood by degrading the low-density lipoprotein receptor (LDLR) that transports LDL-C [7]. In recent years, PCSK9 is one of the promising hot targets in the field of cardiovascular research [8], and the PCSK9 antibody has been used to treat some patients with familial hypercholesterolemia and statin tolerance [9–12]. Qiu et al. [13] found genetic polymorphisms of PCSK9 associated with cardiovascular disease. Several studies have demonstrated that polymorphisms of PCSK9 was associated with the plasma lipid levels in Chinese [14, 15].

Interestingly, it has been found that PCSK9 is also associated with the biological processes of glucose metabolism. Saavedra et al. [16] found that the incidence of diabetes and prediabetes was twice as high in familial hypercholesterolemia (FH) individuals carrying the PCSK9-InsLEU gene mutation as in non-carriers. Mohamed et al. demonstrated that PCSK9 relative expression levels and the E670G (rs505151) AG genotype are cardiovascular disease risk factors among Egyptian diabetics [17]. However, the relationship between PCSK9 genetic polymorphisms and T2DM remains unclear. Thus, the current case–control study was designed to explore the possible correlation of PCSK9 gene polymorphisms with T2DM among Uygur Chinese populations in Xinjiang, China.

## Methods

### Subjects

A total of 1882 Uygur participants were recruited randomly from the First Affiliated Hospital of Xinjiang Medical University between January 2015 and December 2019. 662 of these participants were diagnosed with T2DM and assigned to the case group.

Subjects with T2DM were defined as those who had fasting plasma glucose (FPG) ≥ 7.0 mmol/L, or 2-h postload plasma glucose (2hPG) ≥ 11.1 mmol/L, or a prior T2DM diagnosis and/or the use of a diabetes drug [18]. 1220 participants with normal glucose tolerance (NGT) were designated the control group. NGT was defined as FPG < 6.1 mmol/L and 2hPG < 7.8 mmol/L. Subjects with the following conditions were excluded from recruitment: type 1 diabetes mellitus, special types of diabetes, disorders related to abnormal glucose and lipid metabolism, and individuals receiving lipid-lowering medications.

We conducted the study in accordance with the Declaration of Helsinki. Each participant gave written informed consent and explicit permission for pertinent clinical data collection and DNA analyses. The study was approved by the Ethics Committee of the First Affiliated Hospital of Xinjiang Medical University in Xinjiang, China.

### Clinical and demographic data

The following clinical data were collected by two trained professionals: age, sex, body mass index (BMI), systolic blood pressure (SBP), diastolic blood pressure (DBP), smoking history, drinking history, hypertension history. BMI was defined as weight in kilograms divided by the square of the height in meter. The diagnosis of hypertension was established if the subject met any of the following criteria: previous history of hypertension, use of antihypertensive medication, or three resting blood pressure measurement ≥ 140/90 mmHg on at least two separate occasions [19].

### Genotyping

Blood samples were collected from all participants, and genomic DNA was extracted from peripheral blood leukocytes using a whole blood genome extraction kit (Beijing Bioteke Corporation, Beijing, China). We obtained four tag SNPs of PCSK9 (rs11583680, rs2483205, rs2495477, and rs562556) according to Haploview 4.0 software and the International HapMap Project website (http://hapmap.ncbi.nlm.nih.gov/index.html.en) by using minor allele frequency (MAF) ≥ 0.05 and linkage disequilibrium patterns with r^2^ ≥ 0.8 as a cut-off. The four SNPs of the PCSK9 gene were genotyped by improved multiple ligase detection reaction (iMLDR) genotyping assays.

### Laboratory examination

Peripheral venous blood samples (5 mL) were collected in EDTA-containing tubes from all participants following overnight fasting greater than 8 h for biochemical assays. Biochemical variables, including serum concentrations of creatinine (Cr), blood urea nitrogen (BUN), uric acid (UA), fasting blood glucose (FBG), total cholesterol (TC), triglycerides (TG), high-density lipoprotein cholesterol (HDL-C), and low-density lipoprotein cholesterol (LDL-C), were measured by using standard methods in the Department of Clinical Laboratory at the First Affiliated Hospital of Xinjiang Medical University.

### Statistical analysis

The SPSS version 23.0 statistical software for Windows was applied for statistical analysis of the study (SPSS Inc. Chicago, IL, USA). The continuous variables were expressed in means ± standard deviation (SD) and compared by using an independent sample T-test. Comparisons between categorical variables and differences in genotype distribution were performed using the chi-square test. Risk factors were analysed using logistic regression analyses. P < 0.05 was considered statistically significant.

## Results

### General characteristics of subjects

The baseline characteristics of the 662 T2DM and 1220 control subjects are shown in Table 1. The age, BMI, smoking and drinking habits, Cr, BUN, UA, FBG, TG, TC, and LDL-C were significantly higher in patients with T2DM when compared to controls (all *P* < 0.05). Moreover, the HDL-C was lower in patients with T2DM when compared to controls (*P* < 0.001). Oppositely, we did not observe significant differences between patients and controls regarding gender (*P* = 0.286), hypertension (*P* = 0.223), SBP (*P* = 0.567), and DBP (*P* = 0.316).

**Table 1:** Clinical and metabolic characteristics of subjects

Characteristics	Control (n = 1220)	DM (n = 662)	χ^2^or t	*P* value
Age, mean (SD)	53.23 ± 10.63	56.29 ± 10.47	6.002	** < 0.001**
Gender, male (%)	645 (52.9)	367 (55.4)	1.140	0.286
BMI, mean (SD)	25.50 ± 3.98	26.39 ± 3.62	4.785	** < 0.001**
Hypertension, n (%)	587 (48.1)	338 (51.1)	1.487	0.223
SBP, mean (SD)	131.79 ± 21.88	132.40 ± 21.97	0.573	0.567
DBP, mean (SD)	82.83 ± 15.19	82.10 ± 14.95	− 1.003	0.316
Smoking, n (%)	436 (35.7)	276 (41.7)	6.469	**0.011**
Drinking, n (%)	329 (27.0)	276 (41.7)	42.656	** < 0.001**
Cr, mean (SD)	71.84 ± 23.97	79.21 ± 27.80	6.013	** < 0.001**
BUN, mean (SD)	5.02 ± 1.49	5.41 ± 1.81	4.966	** < 0.001**
UA, mean (SD)	290.17 ± 93.71	311.13 ± 94.66	4.618	** < 0.001**
FBG, mean (SD)	4.60 ± 0.56	7.76 ± 2.86	37.408	** < 0.001**
TG, mean (SD)	1.57 ± 1.12	2.18 ± 1.67	9.450	** < 0.001**
TC, mean (SD)	4.34 ± 1.15	4.56 ± 1.36	3.584	** < 0.001**
HDL-C, mean (SD)	1.19 ± 0.55	1.05 ± 0.44	− 5.409	** < 0.001**
LDL-C, mean (SD)	2.90 ± 1.28	3.20 ± 1.38	4.634	** < 0.001**

Data are presented as number of patients (%) or mean standard ± deviation

*Note*: BMI, body mass index; SBP, systolic blood pressure; DBP, diatolic blood pressure; Cr, creatinine; BUN, blood urea nitrogen; GLU, glucose; TG triglyceride; TC, total cholesterol; HDL-C, high-density lipoprotein cholesterol; LDL-C, low-density lipoprotein cholesterol. The bold indicates statistical difference between the two groups and *P* < 0.05

### Association between PCSK9 gene polymorphisms and T2DM

The genotype frequency distributions of the four SNPs in the PCKS9 gene were in Hardy–Weinberg equilibrium (all *P* > 0.05). The distributions of the SNPs in the PCSK9 gene were indicated in Table 2. In order to intuitively display the distribution of different models of the four SNPs, we have drawn Additional file 1: Figures S1–S4 for viewing. For rs2483205, the distribution of the genotypes, the dominant model (CC vs CT + TT) and the overdominant model (CC + TT vs CT) showed significant differences between the case and control groups (*P* = 0.011 and *P* = 0.041 respectively). For rs2495477, the distribution of the genotypes in the dominant model (AA vs GA + GG) showed significant differences between the two groups (*P* = 0.024). However, there were no significant differences between the two groups in the distributions of rs11583680 and rs562556 SNPs (all *P* > 0.05).

**Table 2:** Distribution of SNPs of PCSK9 gene in T2DM patients and control subjects

Genotype	Model		Case (n, %)	Control (n, %)	χ^2^	*P* value
rs11583680	Codominant	CC	534 (80.7)	977 (80.1)	0.166	0.920
		CT	119 (18.0)	224 (18.4)		
		TT	9 (1.4)	19 (1.6)		
	Dominant	CC	534 (80.7)	977 (80.1)	0.092	0.762
		CT + TT	128 (19.3)	243 (19.9)		
	Recessive	CC + CT	653 (98.6)	1201 (98.4)	0.115	0.735
		TT	9 (1.4)	19 (1.6)		
	Overdominant	CC + TT	543 (82.0)	996 (81.6)	0.043	0.836
		CT	119 (18.0)	224 (18.4)		
rs2483205	Codominant	CC	357 (53.9)	583 (47.8)	6.523	**0.038**
		CT	257 (38.8)	533 (43.7)		
		TT	48 (7.3)	104 (8.5)		
	Dominant	CC	357 (53.9)	583 (47.8)	6.473	**0.011**
		CT + TT	305 (46.1)	637 (52.2)		
	Recessive	CC + CT	614 (92.7)	1116 (91.5)	0.938	0.333
		TT	48 (7.3)	104 (8.5)		
	Overdominant	CC + TT	405 (61.2)	687 (56.3)	4.173	**0.041**
		CT	257 (38.8)	533 (43.7)		
rs2495477	Codominant	AA	340 (51.4)	560 (45.9)	5.334	0.069
		GA	274 (41.4)	554 (45.4)		
		GG	48 (7.3)	106 (8.7)		
	Dominant	AA	340 (51.4)	560 (45.9)	5.123	**0.024**
		GA + GG	322 (48.6)	660 (54.1)		
	Recessive	AA + GA	614 (92.7)	1114 (91.3)	1.181	0.277
		GG	48 (7.3)	106 (8.7)		
	Overdominant	AA + GG	388 (58.6)	666 (54.6)	2.815	0.093
		GA	274 (41.4)	554 (45.4)		
rs562556	Codominant	AA	613 (92.6)	1097 (89.9)	3.744	0.154
		GA	48 (7.3)	121 (9.9)		
		GG	1 (0.2)	2 (0.2)		
	Dominant	AA	613 (92.6)	1097 (89.9)	3.712	0.054
		GA + GG	49 (7.4)	123 (10.1)		
	Recessive	AA + GA	661 (99.8)	1218 (99.8)	0.004	0.947
		GG	1 (0.2)	2 (0.2)		
	Overdominant	AA + GG	614 (92.7)	1099 (90.1)	3.735	0.053
		GA	48 (7.3)	121 (9.9)		

*Note:* The bold indicates statistical difference between the two groups and *P* < 0.05

Multivariate logistics regression showed that, after adjusting for confounding factors such as age, BMI, smoking, drinking, Cr, BUN, UA, FBG, TG, TC, LDL-C, and HDL-C, the SNPs of rs2483205 and rs2495477 were still independent risk factors for T2DM. [for rs2483205 CC vs CT + TT: odds ratio (OR) = 1.321, 95% confidence interval (CI) = 1.078–1.617, *P* = 0.007, Table 3; CC + TT vs CT: OR = 1.255, 95% CI 1.021–1.542, *P* = 0.031, Table 4; for rs2495477 AA vs GA + GG: OR = 1.297, 95% CI 1.060–1.588, *P* = 0.012, Table 5].

**Table 3:** Results of logistic analysis (the dominant model of rs2483205)

Variants	Factors	B	S.E	Wald	*P* value	OR	95% CI
rs2483205	CC versus CT + TT	0.278	0.103	7.234	**0.007**	1.321	1.078–1.617
	Age	0.032	0.005	38.949	0.000	1.033	1.022–1.043
	Gender	0.170	0.122	1.957	**0.162**	1.185	0.934–1.504
	BMI	0.037	0.014	6.984	0.008	1.037	1.010–1.066
	Hypertension	0.189	0.109	2.994	0.084	1.208	0.975–1.496
	Smoking	0.003	0.125	0.001	0.980	1.003	0.786–1.281
	Drinking	− 0.593	0.123	23.408	0.000	0.553	0.435–0.703
	TG	0.315	0.046	46.293	0.000	1.371	1.252–1.501
	TC	− 0.036	0.049	0.536	0.464	0.965	0.876–1.062
	HDL-C	− 0.612	0.136	20.423	0.000	0.542	0.416–0.707
	LDL-C	0.301	0.047	40.430	0.000	1.351	1.231–1.482

*Note:* The bold indicates statistical difference between the two groups and *P* < 0.05

**Table 4:** Results of logistic analysis (the overdominant model of rs2483205)

Variants	Factors	B	S.E	Wald	*P* value	OR	95% CI
rs2483205	CC + TT versus CT	0.227	0.105	4.654	**0.031**	1.255	1.021–1.542
	Age	0.032	0.005	39.699	0.000	1.033	1.023–1.044
	Gender	0.166	0.121	1.861	**0.172**	1.180	0.930–1.498
	BMI	0.036	0.014	6.732	0.009	1.037	1.009–1.065
	Hypertension	0.191	0.109	3.069	0.080	1.210	0.978–1.499
	Smoking	0.002	0.125	0.000	0.988	1.002	0.785–1.279
	Drinking	− 0.589	0.122	23.130	0.000	0.555	0.437–0.705
	TG	0.315	0.046	46.311	0.000	1.371	1.252–1.501
	TC	− 0.037	0.049	0.578	0.447	0.963	0.875–1.061
	HDL-C	− 0.608	0.135	20.132	0.000	0.545	0.418–0.710
	LDL-C	0.301	0.047	40.533	0.000	1.351	1.231–1.482

*Note*: OR, odds ration; CI, confidence interval. The bold indicates statistical difference between the two groups and *P* < 0.05

**Table 5:** Results of logistic analysis (rs2495477)

Variants	Factors	B	S.E	Wald	*P* value	OR	95% CI
rs2495477	AA versus GA + GG	0.260	0.103	6.355	**0.012**	1.297	1.060–1.588
	Age	0.032	0.005	39.410	0.000	1.033	1.023–1.043
	Gender	0.171	0.122	1.990	**0.158**	1.187	0.935–1.506
	BMI	0.036	0.014	6.917	0.009	1.037	1.009–1.066
	Hypertension	0.186	0.109	2.913	0.088	1.205	0.973–1.492
	Smoking	0.003	0.125	0.000	0.984	1.003	0.785–1.280
	Drinking	− 0.596	0.123	23.635	0.000	0.551	0.433–0.701
	TG	0.318	0.046	47.058	0.000	1.374	1.255–1.505
	TC	− 0.036	0.049	0.541	0.462	0.965	0.876–1.062
	HDL-C	− 0.604	0.135	20.098	0.000	0.547	0.420–0.712
	LDL-C	0.297	0.047	39.576	0.000	1.346	1.227–1.476

*Note*: OR, odds ration; CI, confidence interval. The bold indicates statistical difference between the two groups and *P* < 0.05

## Discussion

This was the first study to investigate associations between the common allelic variants in the PCSK9 gene and T2DM in the Chinese Uygur population. Our results showed that rs2483205 and rs2495477 variations in the PCSK9 gene were significantly associated with T2DM susceptibility.

The human PCSK9 gene is located on chromosome 1p32 and is 3617 bases in length, encoding 12 exons [20]. Previous studies have confirmed the essential role of the PCSK9 gene in lipid metabolism. PCSK9 binds to the EGF-A binding domain of the LDLR and affects LDL-C levels via both intracellular and extracellular pathways [21]. It further reduces the clearance of LDL-C by the liver and peripheral organs, with a concomitant rise in circulating LDL-C [22, 23]. There are multiple sequence variations in the human PCSK9 gene, and these variations result in different phenotypes of the body. Polymorphic sites that affect PCSK9 gene function are divided into gain-of-function (GOF) and loss-of-function (LOF), and GOF mutations can lead to increased PCSK9 activity, reduce the number of LDLR in the cell membrane, and lead to increased LDL-C levels in the plasma, while LOF mutations will play the opposite role and be associated with lower plasma levels of LDL-C and reduced risk of cardiovascular disease [24–26].

With the deepening of PCSK9 research, its function in glucose metabolism has been gradually discovered. Multiple epidemiological surveys have shown that circulating PCSK9 concentrations are positively associated with glycemic parameters and T2DM risk [27]. Sterol regulatory element-binding protein-1c (SREBP-1c), HNF-α are important transcription factors of PCSK9. Insulin can induce PCSK9 expression by positively regulating SERBP-1C and inhibit PCSK9 expression by negatively regulating HNF-α [28–30]. In patients with T2DM, PCSK9 expression is upregulated by insulin resistance and subsequent hyperinsulinemia [29, 31]. Inflammatory response promotes the development of T2DM by reducing insulin production and sensitivity [32–34]. While PCSK9 can induce a variety of cells and tissues to secretes pro-inflammatory factors, it is therefore speculated that PCSK9 may be involved in the pathogenesis of T2DM by promoting an inflammatory response [35, 36]. Some studies are supporting those loss-of-function genetic variants of the PCSK9 gene are associated with increased risk of T2DM [37]. It may be because PCSK9 deficiency can lead to increased expression of LDLR in pancreatic β-cells which leads to accumulation of cholesteryl esters, inhibition of islet function and insulin secretion, and causes elevated blood glucose [38].

In the present study, we genotyped polymorphisms of four SNPs in the PCSK9 gene and found that rs2483205 and rs2495477 were associated with T2DM. Rs2495477 is located in the fifth intron of the PCSK9 gene and affects the splicing process of RNA, which may lead to reduced PCSK9 mRNA levels [39]. Rs2483205 is also located in the intronic region of the PCSK9 gene and regulates the promoter flanking regions. It has been shown that rs2483205 is associated with decreased LDL cholesterol concentrations [40]. The CC genotype and CC + TT genotype of rs2483205, the rs2495477 AA genotype were very common in the T2DM patients compared with the control subjects in the Uygur population in Xinjiang. After adjusting for confounders, the rs248320 CC, the rs2483205 CC + TT, and the rs2495477 AA were still independent risk factors for T2DM. We hypothesize that rs2483205 and rs2495477 polymorphisms may be involved in the development of diabetes by decreasing plasma PCSK9 levels, leading to impaired islet function.

There are some limitations to the study. Firstly, our study population was limited to the First Affiliated Hospital of Xinjiang Medical University and may have suffering some risk factors of T2DM. Secondly, as this was an observational study, we cannot definitively establish cause and effect. Thirdly, the present study lacked functional validation of studied SNPs, additional studies need to be conducted to demonstrate the molecular mechanism between PCSK9 gene polymorphisms and T2DM.

## Conclusions

In conclusion, our study suggests that genetic polymorphisms in the PCSK9 gene are associated with T2DM in Uygur subjects in Xinjiang. Subjects with CC genotype or CC + TT genotype of rs2483205 as well as subjects with AA genotype of rs2495477 were associated with an increased risk of T2DM. However, the mechanisms that may link PCSK9 gene polymorphisms to T2DM remain unclear.

OR, odds ration; CI, confidence interval

## Supplementary Information

**Additional file 1: Figure S1.** The distribution of four SNPs genotypes in the case group and control group.

## Data Availability

All data generated or analyzed during this study are included in this published article.

## Abbreviations

T2DM: Type 2 diabetes mellitus
PCSK9: Proprotein convertase subtilisin/kexin type 9
SNPs: Single nucleotide polymorphisms
SBP: Systolic blood pressure
DBP: Diatolic blood pressure
UA: Uric acid
FBG: Fasting blood glucose
NGT: Normal glucose tolerance
iMLDR: Improved multiple ligase detection reaction
SREBP-1c: Sterol regulatory element-binding protein-1c
BMI: Body mass index
Cr: Creatinine
BUN: Blood urea nitrogen
GLU: Glucose
TG: Triglyceride
TC: Total cholesterol
HDL-C: High-density lipoprotein cholesterol
LDL-C: Low-density lipoprotein cholesterol
LDLR: Low-density lipoprotein receptor
