# Supplementary material for: Association of genetic polymorphisms of PCSK9 with type 2 diabetes in Uygur Chinese population
Source: BMC Cardiovasc Disord. 2022 Jun 22;22:284. doi: 10.1186/s12872-022-02710-w (PMC9219175; doi:10.1186/s12872-022-02710-w)
Supplement: Supplementary file 1 — Additional file 1: Figure S1. The distribution of four SNPs genotypes in the case group and control group. [file 12872_2022_2710_MOESM1_ESM.docx]

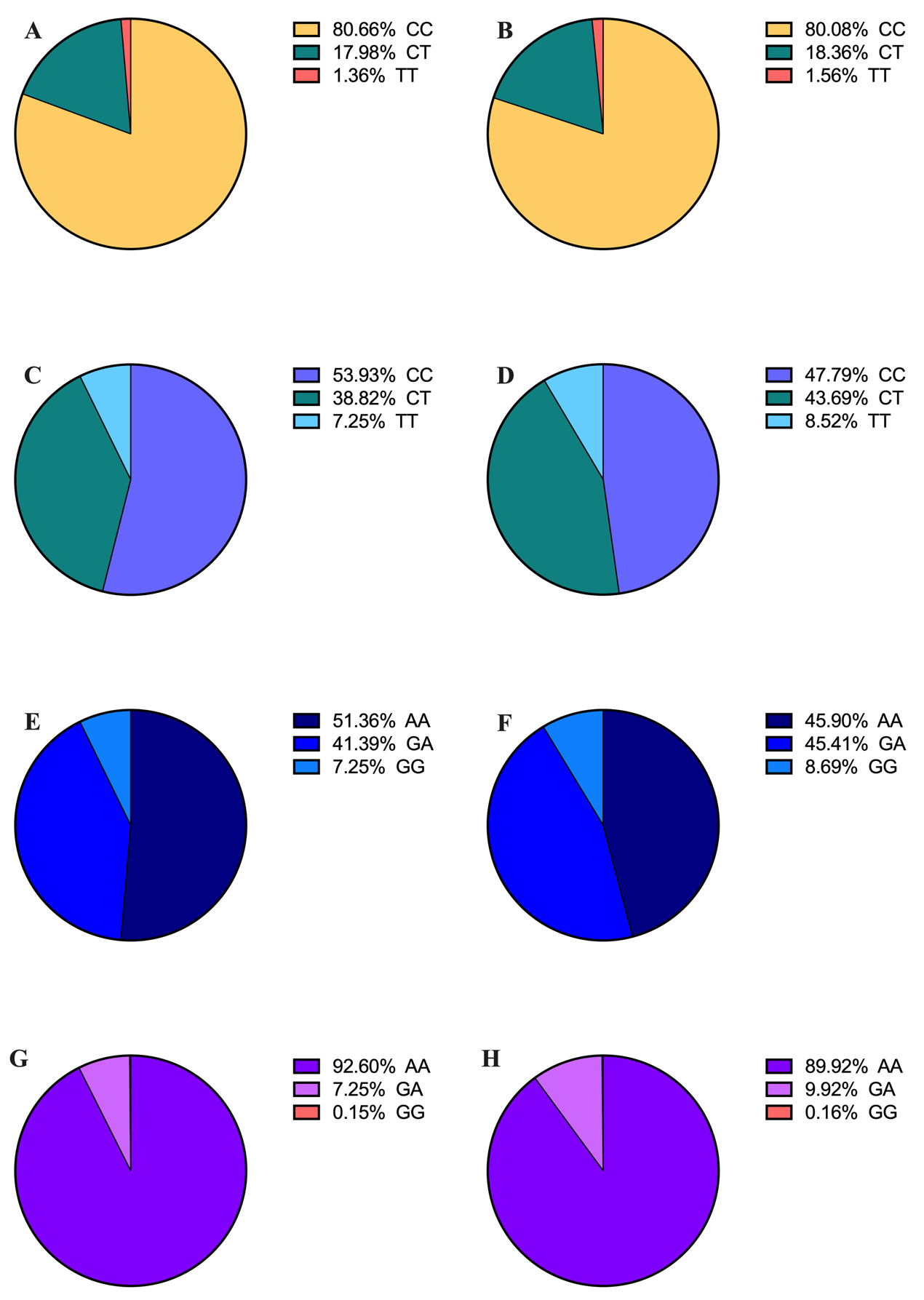


**Figure S1 The distribution of four SNPs genotypes in the case and control group.** A and B: rs11583680 genotypes in case and control group. C and D: rs2483205 genotypes in case and control group. E and F: rs2495477 genotypes in case and control group. G and H: rs562556 genotypes in case and control group.


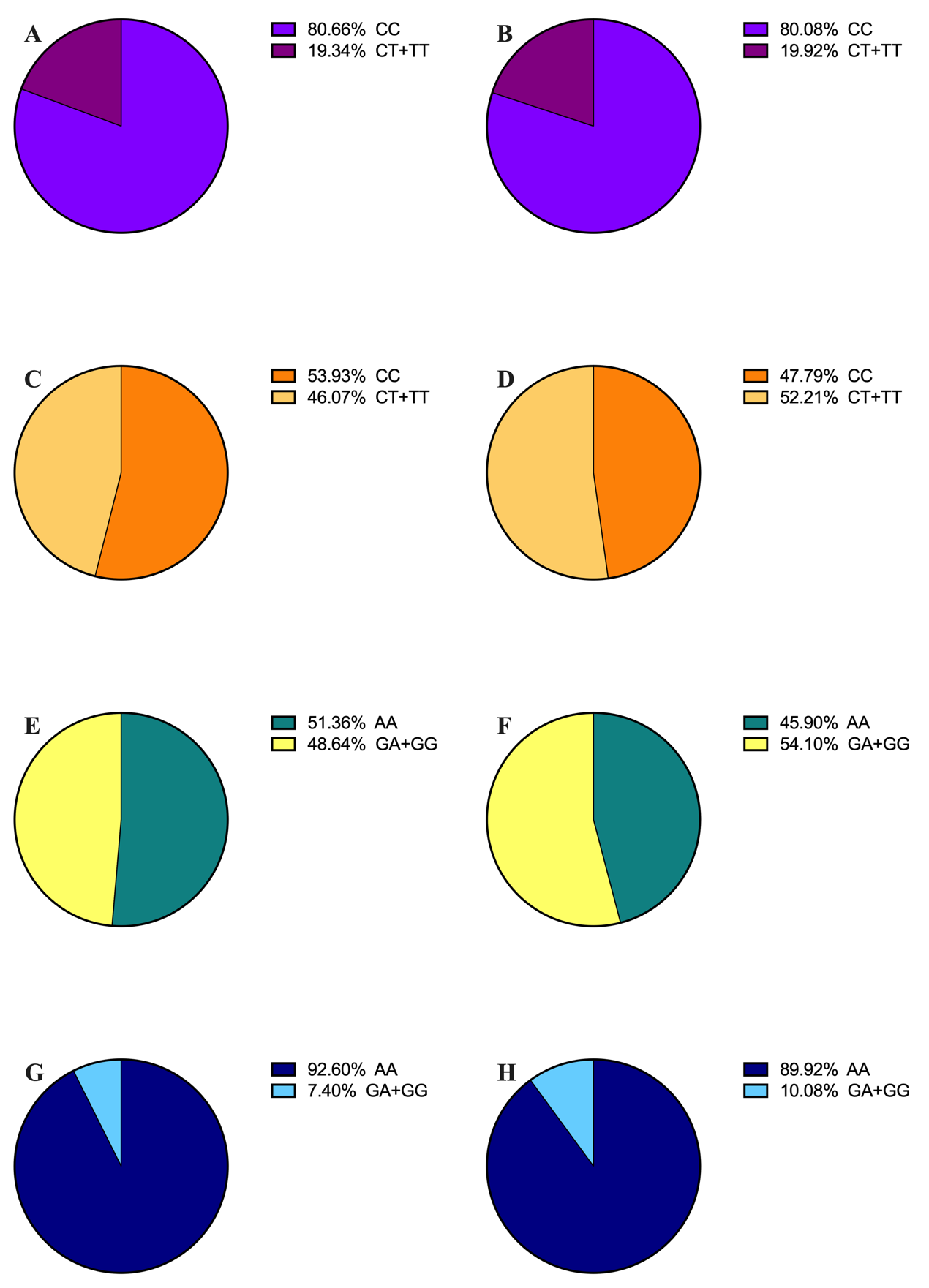


**Figure S2 Distribution of dominant models of four SNPs in cases and controls.**

A and B: The proportion of rs11583680 dominant model in case and control group. C and D: The proportion of rs2483205 dominant mode in case and control group. E and F: The proportion of rs2495477 dominant mode in case and control group. G and H: The proportion of rs562556 dominant mode in case and control group.


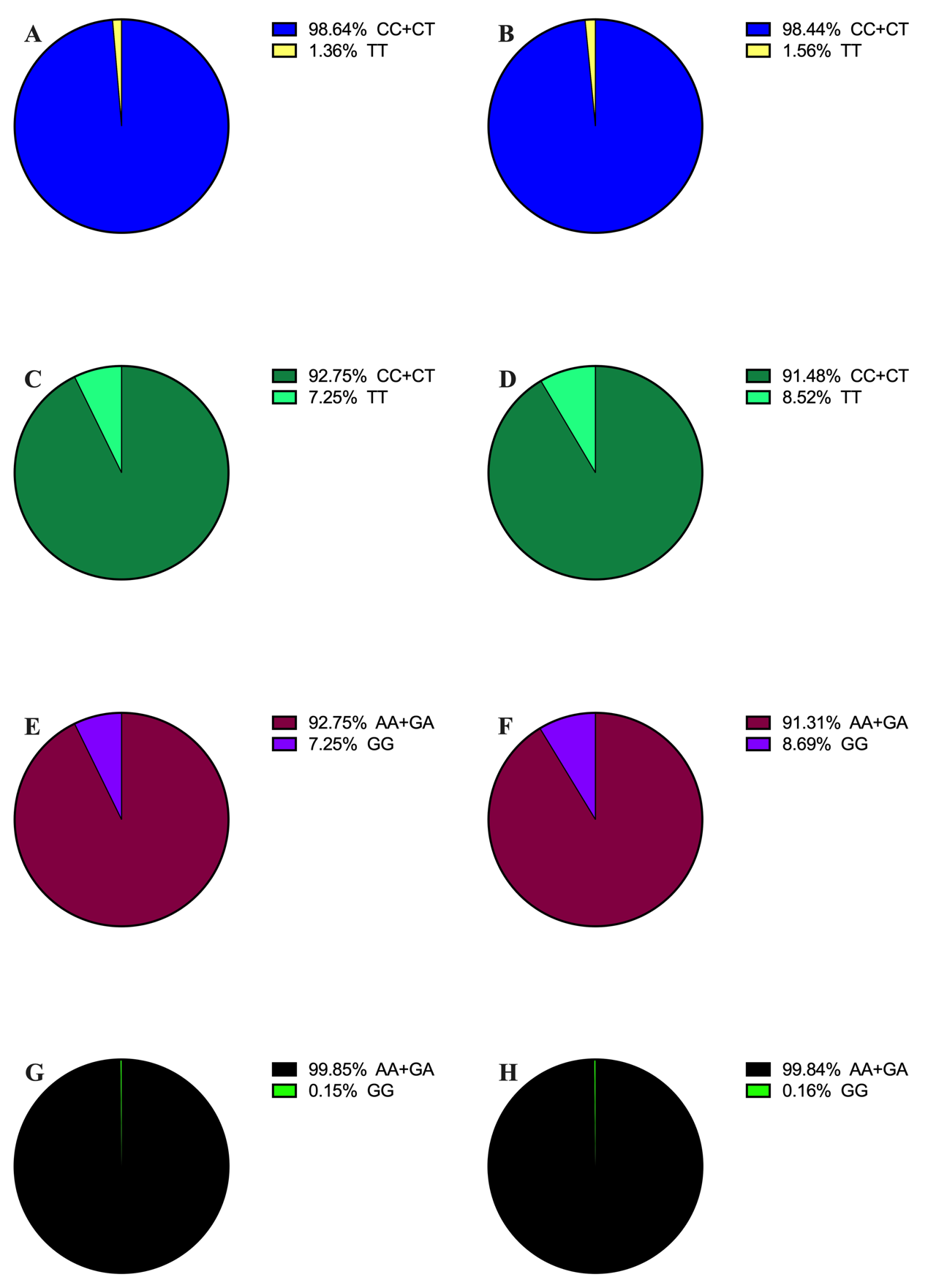


**Figure S3 Distribution of recessive models of four SNPs in cases and controls.**

A and B: The proportion of rs11583680 recessive model in case and control group. C and D: The proportion of rs2483205 recessive mode in case and control group. E and F: The proportion of rs2495477 recessive mode in case and control group. G and H: The proportion of rs562556 recessive mode in case and control group.


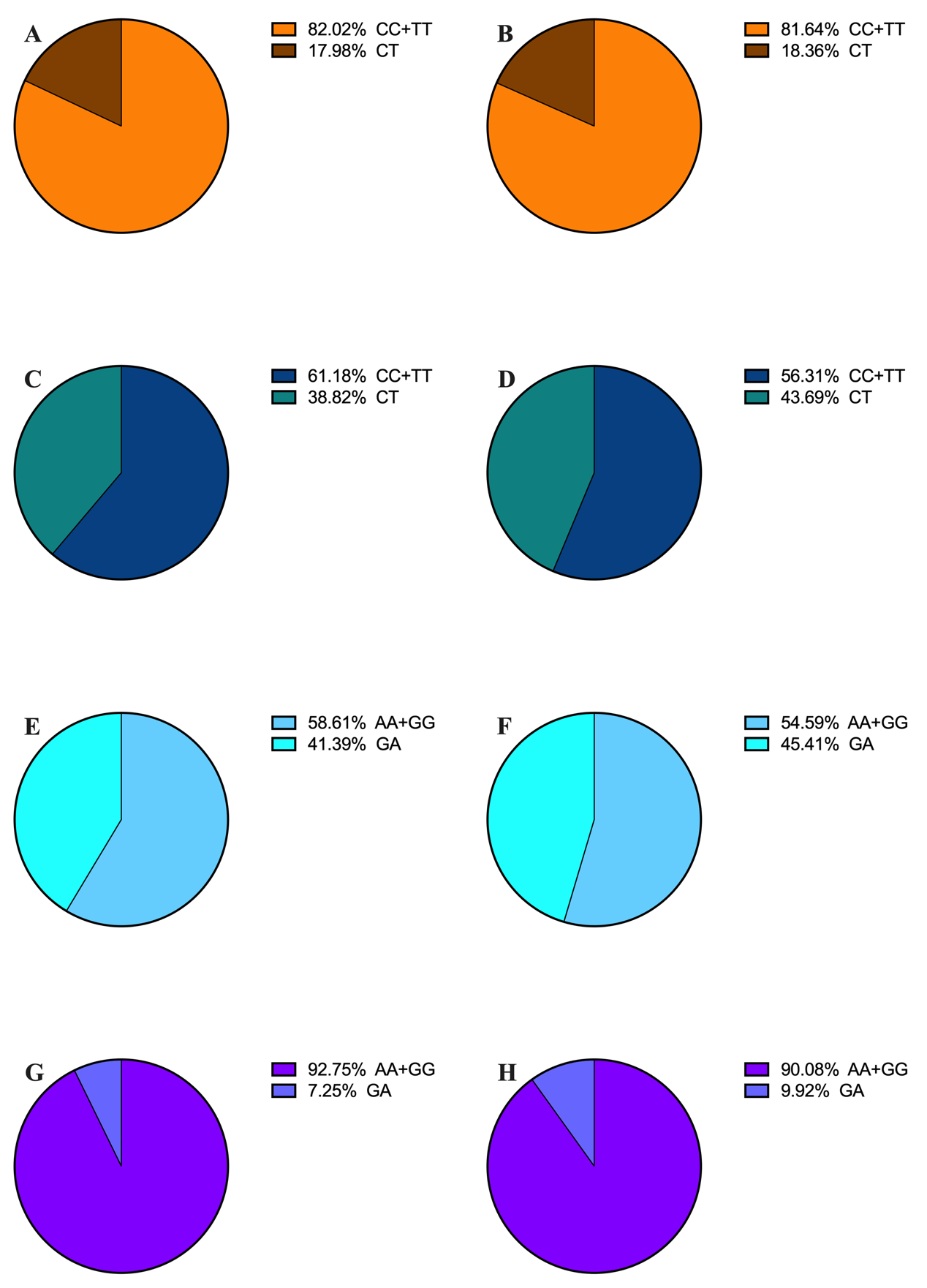


**Figure S4 Distribution of overdominant models of four SNPs in cases and controls.**

A and B: The proportion of rs11583680 overdominant model in case and control group. C and D: The proportion of rs2483205 overdominant mode in case and control group. E and F: The proportion of rs2495477 overdominant mode in case and control group. G and H: The proportion of rs562556 overdominant mode in case and control group.
